# Mood Disturbances Following Traumatic Brain Injury: A Case Report and Treatment Review

**DOI:** 10.7759/cureus.117700

**Published:** 2026-10-09

**Authors:** Carson Balen, John Msaddi, Zayd Chishti, Ryan Chi, Katie Balen, Sophie Kim, Michelle Baker

**Affiliations:** 1 College of Medicine, University of South Florida, Tampa, USA; 2 Biochemistry, University of California Los Angeles, Los Angles, USA; 3 Human Biology, University of California San Diego, La Jolla, USA; 4 Psychiatry and Behavioral Sciences, United States Department of Veterans Affairs, Tampa, USA

**Keywords:** depression prevention, depression therapy, major depressive disorder (mdd), management of tbi, mild tbi, tbi models, traumatic brain injury (tbi)

## Abstract

Traumatic brain injury (TBI) is associated with a substantial and persistent risk of depression, often complicated by cognitive, behavioral, and functional changes. We present a 59-year-old woman who developed progressive depressive symptoms, irritability, and short-term memory difficulties following a moderate TBI. Her presentation required careful consideration of multiple potential contributors to her mood symptoms, including a remote history of substance use, psychosocial stressors, and neurologic injury. This case highlights the diagnostic complexity of depression after TBI and reviews the available evidence for treatment approaches in this population.

## Introduction

Traumatic brain injury (TBI) is a leading cause of global morbidity and mortality, affecting millions of individuals annually across all age groups and injury severities. Although acute medical and surgical management has improved substantially over recent decades, TBI is increasingly recognized not as an isolated event but as a chronic condition with consequences that unfold over years. For example, a Danish nationwide cohort study found that TBI of any severity was associated with an increased risk of work instability, an elevated risk of divorce, and lower academic achievement among children [1]. Even patients who return to work after mild TBI often continue to experience postconcussional symptoms, comorbid depression, and measurable productivity loss. Among the many downstream consequences of TBI, psychiatric disturbance is particularly prevalent and disproportionately drives poor functional outcomes, making it a central determinant of long-term recovery [2].

Depression is the most extensively documented psychiatric sequela of TBI, with a risk profile that appears both substantial and durable. A meta-analysis pooling nearly 400,000 participants found that TBI was associated with an approximately 3.29-fold increase in depression risk relative to uninjured comparisons. The elevated risk did not diminish across the follow-up periods studied, suggesting that the risk may persist rather than normalize over time [3]. Similarly, a multicenter study of hospitalized TBI patients found that 53% met criteria for major depressive disorder (MDD), with a prior history of depression serving as a key predictor of post-injury mood pathology [4]. Depression following TBI is also rarely an isolated syndrome, as it frequently accompanies anxiety and aggression and is associated with poorer social functioning, suggesting shared vulnerability across mood and behavioral domains [5]. Beyond its frequency, post-TBI depression carries substantial cognitive and social consequences.

In particular, the severity and functional consequences of post-TBI depression have been characterized in large cohorts. In a prospective study of 1,570 participants from the TBI Model Systems national database, 22% met criteria for minor depression and an additional 26% met criteria for major depression at one year post-injury [6]. A separate 17-center prospective study of 666 TBI outpatients similarly found that 27% met the symptom threshold required for a DSM-V diagnosis of major depressive disorder, with fatigue, irritability, and anhedonia identified as particularly discriminating features of the syndrome. Furthermore, depression severity demonstrated a dose-response relationship with cognitive impairment, physical disability, and reduced societal participation [7]. Together, these findings suggest that post-TBI depression is closely tied to the core physical and social domains impacted by brain injury.

Emerging neuroimaging research has begun to explore what biological changes might accompany post-TBI depression. Several studies have reported dopaminergic alterations, including differences in receptor binding and transporter availability among patients who develop depression or cognitive difficulties after injury [8,9]. Other work using magnetic resonance spectroscopy has also described changes in glutamate and gamma-aminobutyric acid (GABA) levels in the early weeks after mild TBI, with neurotransmitter balance continuing to shift well into the recovery period [10]. Although preliminary, this early evidence suggests that post-TBI mood symptoms may have a biological substrate in addition to their psychosocial context.

Here, we present a case of major depressive disorder with mood and behavioral dysregulation emerging after a traumatic brain injury in a patient with a remote history of substance use. The case illustrates the diagnostic considerations that arise when a premorbid history and a temporally associated new mood syndrome coexist in the same patient. Additionally, the encounter adds to a still limited body of literature directly linking TBI to new-onset or reemergent mood symptoms.

## Case presentation

A 59-year-old woman with a history of methamphetamine and opioid use presented to an inpatient psychiatric unit via emergency medical services after reporting active suicidal ideation with plans to step in front of moving traffic. She reported that her suicidal ideation had persisted for several months prior to admission. Nine months earlier, she had sustained a TBI in an electric bike accident, with loss of consciousness and a four-day hospitalization. Per outside hospital records, neuroimaging demonstrated a moderate intraparenchymal hemorrhage, which was managed conservatively without surgical intervention. She had no subsequent seizures and received no formal rehabilitation after the injury.

In the immediate recovery period, she had lived independently and worked as a cook, with limited social support, including no spouse or children. However, the patient had two brothers who participated in her initial recovery process. Per their account, within months of the injury, they noted the onset of persistent depressed mood, anhedonia, and emotional lability. Reportedly, she increasingly remained at home and was less socially engaged than her premorbid baseline. No prior suicide attempts were reported, and her family denied any prior mental health difficulties or diagnoses. They reported a history of methamphetamine and opioid use approximately weekly for over a decade in the patient but endorsed abstinence for several years.

On examination, she was cooperative and coherent, with a depressed mood and restricted affect. Her thought process was organized, and there was no evidence of psychosis. Her score on the Montreal Cognitive Assessment (MoCA), a 30-point cognitive screening tool, was 28/30, with points lost on delayed recall, although collateral history corroborated ongoing short-term memory difficulties not captured by the screening measure [11].

Neurologic examination was grossly nonfocal, with no new lateralizing signs or cranial nerve abnormalities, and there was no headache, seizure activity, or acute change in alertness to suggest an evolving intracranial process. Initial laboratory results were unremarkable, including a negative urine drug screen and complete blood count, as shown in Tables 1, 2. She was started on amlodipine 5 mg daily for consistently elevated blood pressure noted during admission. The patient had no prior knowledge of a hypertension diagnosis, consistent with the chart review. 

**Table 1:** Urine Drug Screen on Admission POC: point of care; MDMA: 3,4-methylenedioxymethamphetamine.

Parameter/drug tested	Result
POC barbiturates	Negative
POC benzodiazepine	Negative
POC buprenorphine	Negative
POC cocaine	Negative
POC marijuana	Negative
POC MDMA	Negative
POC methadone	Negative
POC amphetamine	Negative
POC morphine	Negative
POC opiates	Negative
POC oxycodone	Negative
POC phencyclidine	Negative
POC tricyclic antidepressants	Negative
POC fentanyl	Negative

**Table 2:** Complete Blood Count on Admission WBC, white blood cell count; RBC, red blood cell count; Hgb, hemoglobin; HCT, hematocrit; MCV, mean corpuscular volume; MCH, mean corpuscular hemoglobin; MCHC, mean corpuscular hemoglobin concentration; RDW, red cell distribution width; MPV, mean platelet volume; µL, microliter; g/dL, grams per deciliter; fL, femtoliter; pg, picogram.

Test	Result	Reference Range
WBC	7.8 × 10³/µL	4.4–10.5 × 10³/µL
RBC	3.79 × 10⁶/µL	3.75–5.00 × 10⁶/µL
Hemoglobin	12.8 g/dL	11.4–14.7 g/dL
Hematocrit	38.8%	34.3–45.5%
MCV	90.4 fL	80.5–99.8 fL
MCH	32.8 pg	26.8–33.0 pg
MCHC	33.0 g/dL	31.0–35.4 g/dL
RDW	13.1%	11.7–14.7%
Platelet	249 × 10³/µL	139–361 × 10³/µL
MPV	9.3 fL	8.9–12.7 fL

Given the temporal relationship between her TBI and the subsequent onset of depression, irritability, and cognitive complaints, the primary clinical formulation was a mood disorder due to known TBI. Acute intracranial events were ruled out based on stable neurological examinations, and a substance-induced etiology was excluded given verified abstinence based on multiple collateral reports and negative toxicology.

Treatment during admission was multimodal, combining pharmacologic and psychotherapeutic approaches. Oral sertraline was initiated at 50 mg daily and gradually titrated to 100 mg daily over two weeks. Concurrently, she engaged in daily individual cognitive behavioral therapy targeting depressive cognitions, supportive therapy focused on post-injury role adjustment, and group therapy centered on coping mechanisms.

During the first week of treatment, the patient exhibited minimal clinical change, consistent with the expected latency of selective serotonin reuptake inhibitors (SSRIs). By the second week, parallel to sertraline dose optimization and active psychotherapy engagement, she demonstrated marked clinical improvement. Serial depression rating scales reflected a steady reduction in symptom severity.

By the time of discharge, approximately two weeks after admission, the patient reported significant improvement in mood, denied passive or active suicidal ideation, and displayed a future-oriented outlook. She tolerated sertraline without adverse effects. At discharge, she was psychiatrically stable on sertraline 100 mg daily and amlodipine 5 mg daily, with psychiatric and supportive follow-up arranged.

## Discussion

This patient received cognitive behavioral therapy, supportive psychotherapy, and group-based treatment during her hospitalization, situating her care within a growing literature on behavioral interventions for post-TBI depression. In a randomized trial directly comparing cognitive behavioral therapy to supportive psychotherapy for depression after TBI, both treatment arms produced significant reductions in depressive symptoms, with neither modality demonstrating superiority over the other [12]. Turning to a different psychotherapeutic approach, a multisite, wait-list-controlled trial of mindfulness-based cognitive therapy found a significantly greater reduction in depressive symptoms in the treatment group, with improvement maintained at three-month follow-up [13]. Across these two trials, psychotherapy consistently produced a measurable reduction in depressive symptoms. Returning to this patient, the lack of longitudinal follow-up limits direct comparison. However, general symptomatic benefit is consistently reported in the literature, providing reassurance for the approach of combining structured psychotherapy with pharmacologic treatment.

Sertraline was the medication chosen for this patient, titrated to 100 mg daily. SSRIs represent the most thoroughly investigated class for TBI-associated depression. A randomized, placebo-controlled trial of sertraline in 80 patients with post-TBI depression found significant improvement in depressive symptoms and quality of life at three, six, and nine months [14]. Furthermore, a separate randomized, placebo-controlled trial of prophylactic sertraline given in the first three months after moderate-to-severe TBI similarly found significantly fewer depressive symptoms in the sertraline group [15]. Overall, these two studies support sertraline as a helpful option for TBI-associated depression. Accordingly, the patient's dosing and two-week response trajectory mirror these clinical trial findings, though her clinical improvement likely reflects the combined benefit of pharmacotherapy, daily psychotherapy, and the structured inpatient environment.

While psychostimulants were not initiated during this admission, they represent an important alternative or augmenting strategy in patients with TBI-associated cognitive complaints. A comparative trial evaluating methylphenidate, sertraline, and placebo found that methylphenidate achieved antidepressant efficacy equivalent to sertraline while providing additional improvements in cognitive function and fatigue [16]. A separate randomized, placebo-controlled trial reinforced these findings, demonstrating significant reductions in depression scores alongside cognitive gains in post-TBI patients treated with methylphenidate [17]. Across these two trials, methylphenidate was consistently associated with significant improvement in both mood and cognitive symptoms. Returning to this patient, had her short-term memory deficits and executive dysfunction remained functionally disabling despite mood stabilization, methylphenidate augmentation would have served as a logical second-line consideration, though her remote history of methamphetamine use would have warranted careful risk-benefit discussion before initiation.

Neuromodulation represents a newer, nonpharmacologic treatment approach for concussion-associated depression, distinct from the treatment options discussed above. A meta-analysis of ten randomized trials of noninvasive brain stimulation for post-TBI depression found that stimulation was significantly more effective than control, with repetitive transcranial magnetic stimulation (rTMS) showing a significant effect [18]. Consistent with this pooled finding, a randomized controlled trial of low-frequency rTMS to the right dorsolateral prefrontal cortex in patients with TBI found a significant reduction in depression severity in the treatment group compared with sham [19]. Combined, this evidence supports a possible antidepressant benefit for rTMS in TBI. While rTMS was not initiated during this acute stabilization, it remains a relevant option should her symptoms recur or prove resistant to maintenance pharmacotherapy.

Several aspects of this patient's treatment plan align well with what this literature would suggest. Sertraline has a comparatively strong evidence base for concussion-associated depression, and her improvement over the admission is broadly consistent with the positive trials discussed above. Likewise, the multimodal therapeutic approach reflects the general supportive pattern of psychotherapy in this literature. Finally, several alternative treatments presented above, including the use of stimulants and rTMS, remain relevant as possible adjuncts in achieving long-term mood and cognitive stability. This patient's two-week response was notably faster than the responses reported in the therapeutic trials above, though no randomized trial has directly compared combined SSRI and psychotherapy with either treatment alone in TBI-associated depression.

## Conclusions

A recurring limitation across the literature reviewed here is the substantial heterogeneity in injury severity and the variable time interval between injury and trial enrollment. This variation likely obscures clinically meaningful differences between patients with very different injury profiles, so comparisons drawn between individual cases and the studies cited above should be treated as approximate rather than definitive. Relatedly, no randomized trial to date has compared combined SSRI and psychotherapy with either treatment alone in this population, and this patient's relatively rapid two-week response supports further controlled study of this combination. More broadly, neurobehavioral care after TBI sits at the intersection of neurology and psychiatry, requiring clinicians to confirm that a patient's neurological status has been thoroughly evaluated and stabilized before proceeding along standard psychiatric treatment pathways. This patient population also remains somewhat distinct from patients with primary psychiatric disease, and its presentation and treatment response may not always follow the same patterns, underscoring the importance of continued research in the treatment of post-TBI mood disorders.
